# Digital Rhythm Surveillance After Postoperative Atrial Fibrillation in Cancer Survivors: A Cardio-Oncology Survivorship Pathway

**DOI:** 10.3390/medicina62081597

**Published:** 2026-08-20

**Authors:** Sotiris Kyriakou, Georgios P. Georghiou, Panos Georghiou, Argyris Kyriakou, Amalia Georgiou, Marilina Neokleous, Andrew Xanthopoulos, Filippos Triposkiadis

**Affiliations:** 1 Barts and The London School of Medicine and Dentistry, Queen Mary University of London, London EC1M 6BQ, UK; s.kyriakou@nhs.net (S.K.); panos.georghiou@gmail.com (P.G.); 2 Department of Surgery, Gray Faculty of Medical & Health Sciences, Tel Aviv University, Tel Aviv 6997801, Israel; 3 School of Medicine, European University Cyprus, Nicosia 2404, Cyprus; andrewvxanth@gmail.com (A.X.); ftriposkiadis@gmail.com (F.T.); 4 Barking, Havering and Redbridge University Hospitals NHS Trust, London RM7 0AG, UK; argyris.kyriakou6@nhs.net; 5 Krankenhaus Maria Hilf Krefeld, 47805 Duseldorf, Germany; amalia.georgiou.med@gmail.com; 6 School of Medicine, University of Cyprus, Nicosia 2029, Cyprus; mneokl01@ucy.ac.cy

**Keywords:** postoperative atrial fibrillation, cancer survivors, wearable ECG, remote monitoring, cardio-oncology, digital health, rhythm surveillance, post-discharge care, anticoagulation

## Abstract

Postoperative atrial fibrillation (POAF) after cardiac surgery is often managed as a transient inpatient arrhythmia, yet recurrent or silent atrial fibrillation (AF) after discharge may identify persistent atrial vulnerability. This issue is particularly relevant in cancer survivors, whose thrombotic and bleeding risks vary according to cancer activity, treatment exposure, thrombocytopenia, frailty and planned procedures. For this narrative review, PubMed/MEDLINE and Scopus were searched from inception to 16 June 2026, with Google Scholar used for supplementary citation tracking. A total of 50 publications were included in the final narrative synthesis. Direct evidence at the intersection of cancer, cardiac surgery, POAF and digital monitoring remains limited. Accordingly, the proposed “Digital Cancer-POAF Survivorship Pathway” is presented as a conceptual, hypothesis-generating framework, rather than a validated clinical algorithm. Its distinctive contribution is to connect cancer-state phenotyping and treatment-specific arrhythmic risk with post-discharge rhythm surveillance, electrocardiogram (ECG) confirmation, multidisciplinary interpretation, data governance and outcomes for prospective validation. Digital monitoring may increase AF detection; however, whether it reduces stroke, bleeding, readmission, or healthcare utilisation in this population is unknown. Neither a low detected AF burden nor the absence of AF during finite monitoring has been validated as an independent basis for anticoagulation decisions.

## 1. Introduction

Postoperative atrial fibrillation (POAF) remains an important rhythm complication after cardiac surgery. It usually appears in the early stages of recovery and is managed with rate or rhythm control, correction of reversible triggers, and anticoagulation if required. This inpatient approach is often appropriate because many episodes resolve before discharge, while early postoperative care prioritises haemodynamic stability and reversible precipitants. However, the prevailing view of POAF as just a transient reaction to surgical stress is increasingly challenged, as it has been associated with an increased risk of recurrent atrial fibrillation (AF), stroke, readmission and mortality. A normal sinus rhythm upon discharge does not guarantee absence of future risk.

POAF may reflect underlying atrial vulnerability. Inflammation from surgical procedures and electrolyte irregularities can contribute to AF after an operation, but these influences are shaped by the individual circumstances of each patient. Key contributors include age, left atrial enlargement, and surgical complexity, while the impact of current or prior malignancies is often overlooked in cardiac surgical data.

Cancer survivorship has become an important part of modern cardiovascular care. Patients with active or previous cancer increasingly live long enough to develop degenerative valvular disease, coronary artery disease or other indications for cardiac surgery. Nevertheless, the term “cancer survivor” spans clinically dissimilar states. Active cancer receiving treatment, active cancer not currently receiving treatment, recently completed treatment and long-term remission differ in thrombotic risk, bleeding risk, treatment interactions, frailty, prognosis, and potential benefit from surveillance. These categories are defined explicitly in Section 3, and should not be collapsed into a single binary variable.

Post-discharge rhythm monitoring is more than a technical question. In cancer survivors with POAF, the key question is whether monitoring improves clinical decision-making. A brief inpatient telemetry record fails to adequately characterise the AF burden post-discharge, and a routine clinic electrocardiogram (ECG) may overlook paroxysmal or asymptomatic AF. Symptom-driven follow-up is particularly questionable in postoperative and oncology patients, as manifestations such as palpitations, fatigue, dyspnoea, and dizziness may be attributed to anaemia, deconditioning, pain, chemotherapy, infection, or anxiety. Detection is useful only if it changes management [1,2].

This narrative review proposes a practical approach to digital rhythm surveillance in oncology patients with POAF after cardiac surgery and introduces the “Digital Cancer-POAF Survivorship Pathway” as a conceptual, hypothesis-generating model for post-discharge care. Three of the cited publications originate from the present author group and address adjacent but distinct questions. A prospective study of 400 consecutive cardiac surgical patients identified cancer as an independent predictor of in-hospital POAF [3]. A subsequent review examined the epidemiology and clinical implications of that association [2], and a further review addressed the mechanistic basis of cancer-related atrial vulnerability and its perioperative consequences [4]. All three are concerned with predicting or explaining POAF before and during the surgical admission. None addresses what happens after discharge, none examines rhythm monitoring, and none proposes a follow-up structure. The present review begins where those reports finish and asks how post-discharge rhythm surveillance might be organised once POAF has already occurred. Its incremental contribution is therefore the post-discharge frame itself, together with cancer-state phenotyping applied to surveillance decisions, therapy-specific arrhythmic risk including BTK inhibitors, comparison of monitoring modalities, mandatory ECG confirmation, data governance and equity, and a candidate operational protocol with prespecified validation outcomes [5,6,7]. No text, figure or table is reproduced from those publications, and no new patient data are presented here. The framework has not been prospectively tested and should not be interpreted as a treatment recommendation.

## 2. Review Methodology

The core Boolean strategy was (“postoperative atrial fibrillation” OR “post-operative atrial fibrillation” OR POAF) AND (“cardiac surgery” OR CABG OR “coronary artery bypass” OR “valve surgery”) AND (“cancer” OR “malignant” OR “oncology” OR “cardio-oncology”) AND (“wearable” OR “remote monitoring” OR “rhythm surveillance” OR “smartwatch” OR “ECG patch” OR “Holter’ OR “photoplethysmography” OR “implantable loop recorder”). Because literature directly combining all four concepts was sparse, paired searches were also conducted for POAF and post-discharge monitoring, cancer and AF, and cancer therapy and AF. Database syntax was adapted as required. Google Scholar was used only for backward citation tracking, rather than as the source of a quantitative record count.

Eligible sources were English-language adult human studies, systematic reviews and meta-analyses, professional guidelines or consensus statements, diagnostic-accuracy studies, implementation studies and economic evaluations relevant to POAF, cancer-associated AF or digital rhythm monitoring. Paediatric studies, animal or in vitro work, isolated case reports, non-peer-reviewed material and studies without relevance to the review question were excluded.

Database searches identified 330 records: 187 from PubMed/MEDLINE and 143 from Scopus. Both databases were searched from inception to 16 June 2026, with results restricted to English-language publications. An additional 31 potentially relevant records were identified through backward citation tracking of relevant full-text publications, resulting in 361 records overall. After removal of 98 duplicates, 263 unique records underwent title-and-abstract screening, of which 151 were excluded. The full texts of 112 publications were assessed for eligibility, and 62 were excluded because the population fell outside the review scope (*n* = 19), the publication did not address atrial fibrillation, postoperative atrial fibrillation or rhythm surveillance (*n* = 15), the publication was a commentary or editorial without substantive supporting evidence (*n* = 11), the publication did not provide evidence relevant to the clinical questions addressed by the pathway (*n* = 9), or it was a secondary or overlapping report without additional relevant information (*n* = 8). Fifty publications were included in the final narrative synthesis. Screening was undertaken independently by S.K. and G.P.G., with uncertainties resolved through discussion. No protocol was registered and no quantitative meta-analysis was undertaken.

Evidence was prioritised in the following order: studies directly addressing cancer and cardiac-surgery POAF; contemporary AF, POAF and cardio-oncology guidelines; randomised or prospective monitoring studies after cardiac surgery; systematic reviews and large observational cohorts; and, only when direct evidence was unavailable, indirect evidence from broader AF-screening or cancer-associated AF populations. Indirect evidence is identified as extrapolative throughout the review. Reference lists were checked for additional relevant studies, and recent publications and DOI details were verified against PubMed or the publisher record.

## 3. Definitions and Target Population

For this review, index POAF is defined operationally as new-onset AF or atrial flutter occurring after cardiac surgery and before discharge in a patient without documented preoperative AF, confirmed by a 12-lead ECG or an interpretable telemetry tracing lasting at least 30 s, or requiring treatment because of clinical or haemodynamic significance [8]. Studies using other definitions are described according to their original criteria. AF first confirmed after discharge is classified as post-discharge recurrent AF, whereas AF documented before surgery is considered pre-existing or established AF, and lies outside the pathway’s primary target population. The term “apparently transient POAF” denotes restoration of sinus rhythm before discharge; it does not imply that the arrhythmia or its associated risk has resolved.

The target population is divided into four clinically meaningful cancer states (Table 1). This classification is pragmatic rather than validated, and should be supplemented by tumour type and stage, prognosis, frailty, thrombocytopenia, renal and hepatic function, previous thoracic radiotherapy, systemic-therapy exposure, treatment interactions, and anticipated procedures. A fixed time boundary for “recently completed treatment” has not been validated; future studies should prespecify and justify the interval used.

## 4. POAF After Cardiac Surgery: From Inpatient Arrhythmia to Post-Discharge Risk Signal

**Figure 1:**
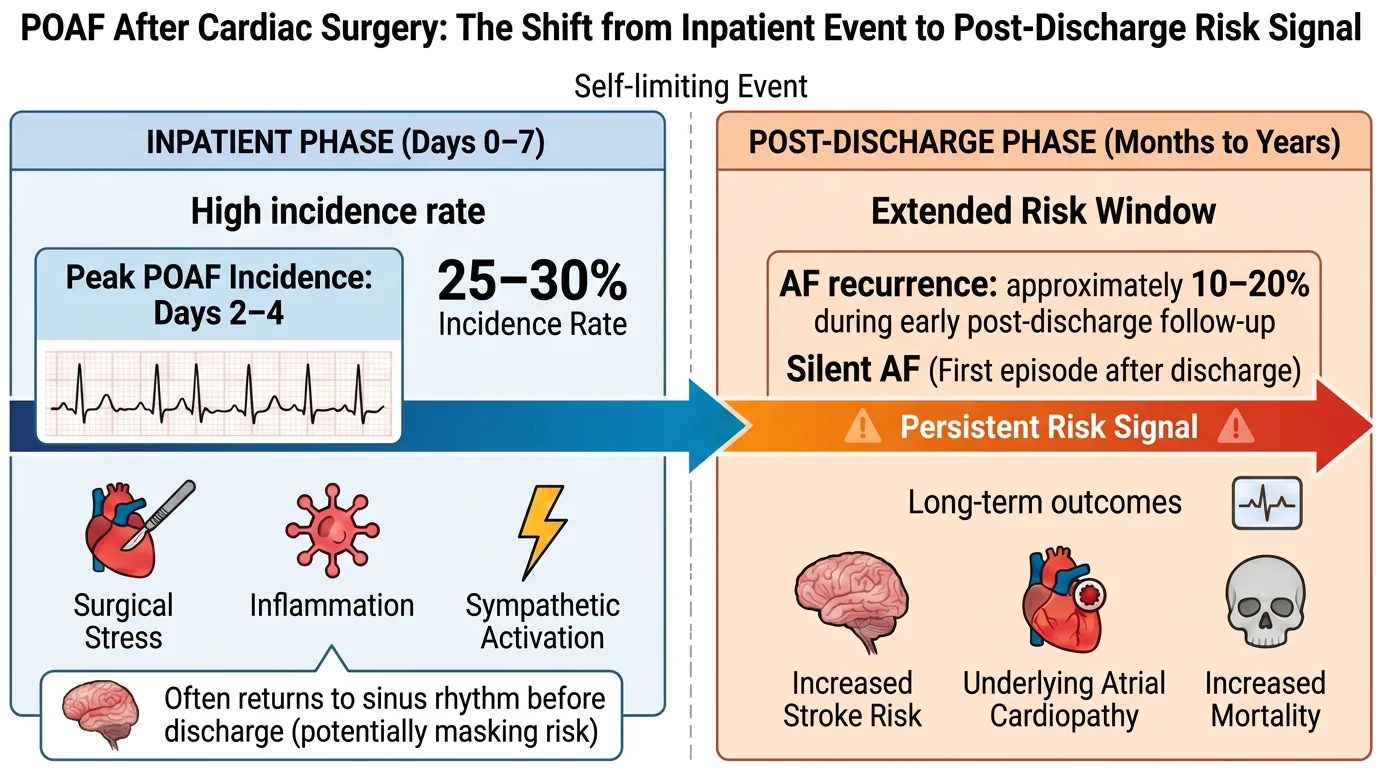
Conceptual shift in postoperative atrial fibrillation (POAF) after cardiac surgery, from a self-limiting inpatient event to a persistent post-discharge risk signal. In the inpatient phase (days 0–7), POAF occurs in approximately 25–30% of patients, peaks on days 2–4, and is driven by surgical stress, inflammation and sympathetic activation; most episodes revert to sinus rhythm before discharge, potentially masking residual risk. In the post-discharge phase (months to years), recurrent or silent AF marks an extended risk window associated with increased stroke risk, underlying atrial cardiopathy and higher mortality. AF, atrial fibrillation; POAF, postoperative atrial fibrillation.

The prospective VISION Cardiac Surgery cohort provides contemporary context: among 12,234 patients, POAF occurred in 31.8%, somewhat higher than estimates from older series; clinical AF between 30 days and 1 year was detected in 6.9% of patients with POAF compared with 0.6% without POAF, while all-cause death occurred in 3.0% versus 1.7% [9]. In older series, POAF was reported in approximately 25–30% of patients, usually appeared 2–4 days after surgery, and commonly returned to sinus rhythm before discharge [10,11,12]. Historically, it was viewed as a self-limiting recovery-phase arrhythmia, but observational studies and meta-analyses associate it with stroke and mortality [10,11,12]. These associations do not prove that monitoring or rhythm-directed treatment improves outcomes, but they support treating POAF as a post-discharge risk marker, as illustrated in Figure 1. Smaller studies using intensive monitoring report recurrent AF in approximately 10–20% of patients, with detection varying by modality and episode definition [13,14,15]. Sinus rhythm at discharge therefore means only that AF is not present at that moment.

## 5. Cancer-Related Cardiovascular Vulnerability and Therapy-Specific AF Risk

Cancer survivors are a growing and clinically heterogeneous group. The four cancer states defined in Table 1 differ in inflammation, thrombosis, cytopenias, treatment interactions, procedural requirements, frailty and competing prognosis [16]. Consequently, the expected value and possible harms of rhythm surveillance cannot be inferred from a binary history-of-cancer variable.

Anticancer therapies should not be grouped as though they create a uniform arrhythmic risk. Anthracyclines and HER2-directed therapy may promote ventricular dysfunction and secondary atrial remodelling; thoracic radiotherapy can produce late myocardial, pericardial and atrial fibrosis; immune checkpoint inhibitors can cause uncommon but serious myocarditis and conduction or atrial arrhythmias; and selected targeted therapies have more direct associations with AF [16]. Cancer-associated hypercoagulability and systemic inflammation may further modify thrombotic and atrial vulnerability. Treatment exposure, tumour type and stage, laboratory values, frailty, and planned procedures should therefore be reported separately.

Bruton tyrosine kinase (BTK) inhibitors provide an important contemporary example. Ibrutinib has the best-characterised association with AF and also creates management difficulties through hypertension, bleeding and platelet effects, and interactions relevant to anticoagulant and rate- or rhythm-control therapy [17]. In the head-to-head ELEVATE-RR trial, AF or atrial flutter occurred in 9.4% with acalabrutinib and 16.0% with ibrutinib [18]. In the final ALPINE comparison, AF or flutter occurred in 7.1% with zanubrutinib and 17.0% with ibrutinib [19]. These data suggest lower, but not absent, AF risk with newer agents, and do not specifically address patients after cardiac surgery. Decisions to continue, temporarily interrupt or change BTK-inhibitor therapy should therefore be made jointly by haematology/oncology and cardiology; a wearable alert alone should never determine cancer-treatment continuation or anticoagulation.

## 6. Current Evidence Linking Cancer and POAF

Direct data on cancer and POAF after cardiac surgery remain sparse [2,4]. Broader AF literature identifies a bidirectional association between cancer and AF, including treatment-specific associations, but registries often lack treatment timing and cancer granularity [16,17,18,19,20,21]. In the prospective 400-patient study, 66 patients developed POAF and 334 did not. Cancer was recorded in 10/66 patients with POAF (15.2%) and 13/334 without POAF (3.9%), corresponding to an adjusted odds ratio of 3.85 (95% confidence interval 1.54–9.66) [3]. The publication treated cancer as a binary history variable and did not report activity, treatment timing or remission status; subgroup-specific inference is therefore not possible. Contemporary guidelines emphasise balancing thromboembolic and bleeding risks yet provide limited population-specific direction for POAF in cancer survivors [22,23,24]. Moreover, the performance of CHA_2_DS_2_-VASc and HAS-BLED varies by cancer type, with particularly limited discrimination of HAS-BLED for major bleeding; these scores may inform, but should not determine, decisions [25]. No trial has evaluated a complete digital-monitoring strategy specifically in cancer survivors with cardiac-surgery POAF. The rationale for surveillance therefore remains largely extrapolated from general POAF, cancer-associated AF and digital-monitoring studies.

## 7. The Post-Discharge Detection Gap

Many AF episodes are likely to be missed in standard follow-up visits. After telemetry is stopped, a rhythm check is performed when the patient complains of symptoms or when additional monitoring is arranged at clinician discretion or when AF is captured incidentally on ECG [26,27].

This is particularly relevant in cancer survivors, as their care is often spread across cardiac surgery, cardiology, oncology and primary care. The patient may report experiencing palpitations during their oncology visit; however, they may not link fatigue, dizziness, or breathlessness to rhythm disturbance. These symptoms can often be attributed to factors including anaemia, effects from treatments, infections, pain, lack of restful sleep, and delayed recovery from surgical procedures [2,16,28]. A single ECG in an oncology clinic provides only a rhythm snapshot, and intermittent AF may therefore be missed by clinic-based ECG [26].

Digital monitoring can identify AF missed by routine clinic-based follow-up. Available approaches include ambulatory ECG patches, Holter monitoring, handheld ECG, smartwatch ECG and PPG-based alerting [26,27,28,29,30,31]. Detection is not equivalent to clinical benefit: the more difficult question is whether episode duration, frequency, or cumulative burden should change treatment. A monitor-defined AF episode of at least 30 s can serve as a diagnostic or research endpoint, but it is not a validated anticoagulation threshold in this population.

Interpretation is especially difficult in cancer survivors. Brief or device-suspected AF should not automatically be managed as recurrent or established AF. PPG alerts require ECG confirmation, and any confirmed episode should be considered alongside cancer state, platelet count, bleeding history, systemic therapy, drug interactions, and planned procedures. Neither an isolated short episode nor failure to detect AF during finite monitoring establishes the net benefit of anticoagulation [20,32,33].

## 8. Digital Rhythm-Surveillance Technologies

**Figure 2:**
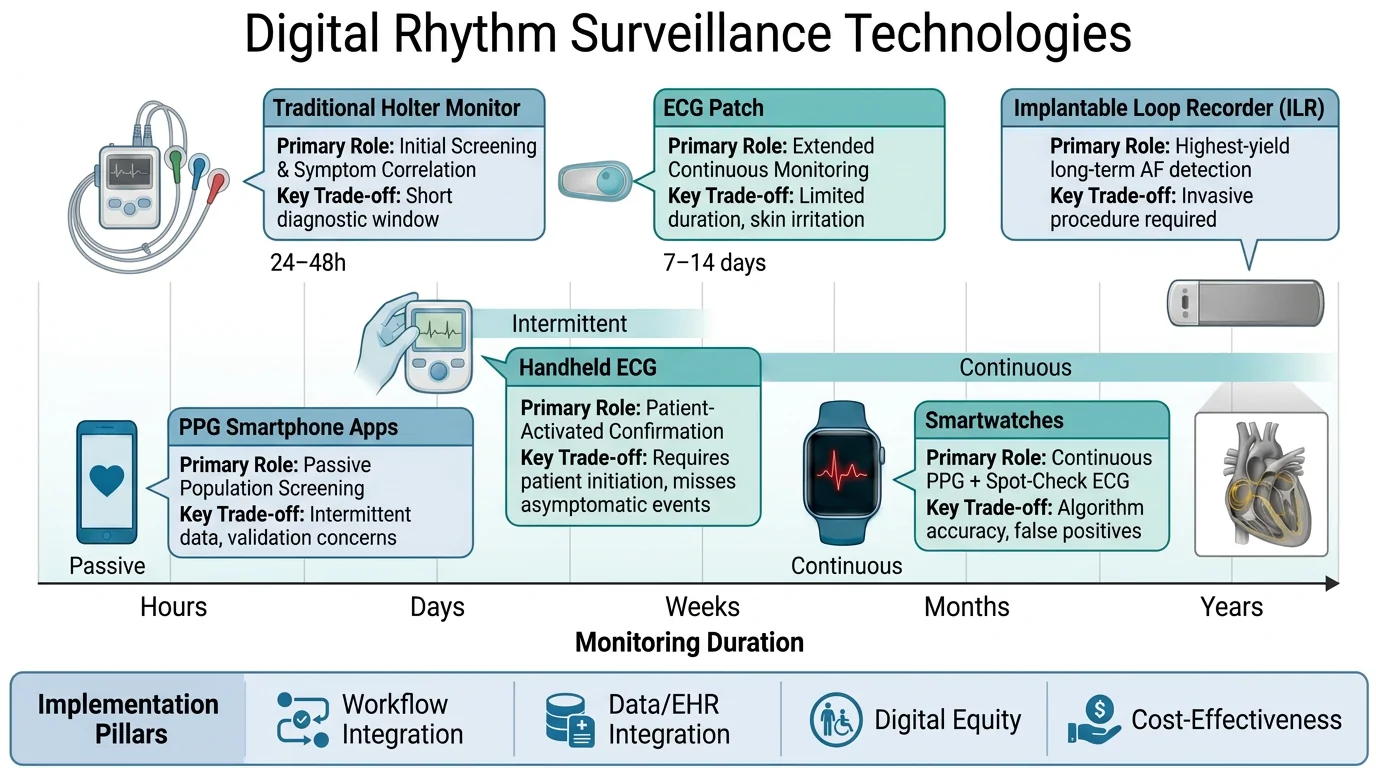
Digital Rhythm-Surveillance technologies positioned by typical monitoring duration (hours to years). For each modality—Holter monitor (24–48 h), ECG patch (7–14 days), implantable loop recorder, handheld single-lead ECG, smartwatch (continuous PPG plus spot-check ECG), and PPG smartphone applications—the primary role and key trade-off are summarised. Four implementation pillars—workflow integration, data/EHR integration, digital equity, and cost-effectiveness—apply across all modalities. ECG, electrocardiogram; EHR, electronic health record; ILR, implantable loop recorder; PPG, photoplethysmography.

Several technologies can extend rhythm surveillance beyond discharge, but they differ in recording duration, diagnostic certainty, patient burden, and workflow implications (Figure 2 and Table 2). Short Holter monitoring is most likely to capture frequent events, whereas continuous ECG patches provide a longer non-invasive sampling window and increase AF detection compared with usual follow-up [15,26,27]. Implantable loop recorders provide the longest observation but are invasive, costly, and investigational for routine POAF surveillance [13,34]. These differences support modality selection according to the clinical question, rather than commercial availability.

Patient-triggered handheld or smartwatch ECG can provide interpretable rhythm strips during symptoms, but intermittent use may miss asymptomatic AF. PPG-enabled watches or smartphone applications can screen passively for pulse irregularity but do not provide a definitive AF diagnosis without an interpretable ECG. Consumer-device ownership, wear time, technical literacy and data-sharing arrangements also affect performance outside research settings [1,29,30,31,35,36,37].

Technical performance is only one barrier. High-sensitivity tools may generate misleading positives that could cause overdiagnosis. Premature atrial contractions, motion artefact, or other non-AF rhythms can imitate AF on PPG or single-lead ECG recordings. Smartphone camera applications show high pooled sensitivity and specificity, but their positive predictive value can be modest, meaning false-positive results may outnumber true positives in low-prevalence screening populations [33]. False-positive alerts could cause unnecessary anxiety, additional clinic visits, unnecessary ECGs, or extra blood tests in post-surgical patients. Conversely, the detection of brief AF episodes raises concerns regarding clinical significance. Treating every brief episode with anticoagulants could expose patients to unnecessary bleeding risk, particularly in cases involving cancer-related low platelet counts. Digital surveillance requires explicit action criteria, including minimum episode duration or cumulative AF burden. These thresholds remain uncertain, and should be prioritised for research.

Data overload is another major barrier. Continuous monitoring can generate numerous strips or alerts, and high alert volumes may delay review, rather than improve care. Algorithmic or AI-supported triage could prioritise recordings, but it should not autonomously diagnose AF or trigger anticoagulation, antiarrhythmic therapy or changes to cancer treatment. Any such system requires external validation in postoperative cancer populations, access to the underlying ECG, subgroup performance and false-negative auditing, version control, clinician confirmation, and explicit accountability for delayed or missed alerts [1,38].

Even when AF is detected accurately, the therapeutic meaning remains uncertain. Guideline-based thromboembolic assessment remains necessary, but no AF-burden threshold has been validated to determine anticoagulation after POAF in cancer survivors [22,23,24,31]. Studies should therefore separate diagnostic endpoints from treatment rules and should report episode duration, cumulative burden and clinical context, without implying that a numerical threshold mandates therapy.

Workflow determines whether monitoring is usable. A pathway should identify the individuals responsible for data analysis, the frequency of these evaluations, the findings that require escalation, and the clinician responsible for treatment decisions. Possible frameworks include nurse-led rhythm-surveillance clinics, triage systems led by cardiac clinicians, cardio-oncology review pathways, or automated notifications sent to electrophysiology teams. Each framework has unique staffing and financial considerations. Remote device-clinic models are established in several healthcare systems, but a corresponding outpatient structure for post-POAF surveillance remains insufficiently developed. A dedicated POAF or cardio-oncology rhythm clinic is appealing, but remains untested [38,39,40].

EHR integration also remains difficult. Most consumer device data are not easily integrated into hospital EHR systems. Without interoperability standards, including Fast Healthcare Interoperability Resources (FHIR)-based approaches for personal health devices, rhythm data may remain confined to patient-facing apps or commercial cloud platforms. This fragmentation limits longitudinal care, audit, governance and clinical accountability. A cardiologist may not see the data, an oncologist may be unaware of recurrent AF, and the discharge summary may not reflect the post-discharge rhythm burden. General Data Protection Regulation (GDPR) and Health Insurance Portability and Accountability Act (HIPAA) considerations also become relevant when patient-generated data are transmitted, stored or reviewed through third-party platforms [5,38,39].

Privacy and security must be built into the pathway from the start. Continuous or repeated rhythm monitoring can generate sensitive health data, including incidental findings such as bradycardia, pauses or non-AF arrhythmias. Patients should be told who can access their data, how often the data will be reviewed, what will happen if an abnormality is detected, and what the limits of monitoring are. Hospitals and device providers also need clear arrangements around data storage, encryption, consent, audit processes, and responsibility for missed or delayed alerts [1,38].

Monitoring may support patient counselling after cardiac surgery when results are explained clearly. However, unexplained alerts may encourage repeated device-checking or concern about benign rhythm irregularities. This is especially relevant in cancer survivors, as they may already be dealing with uncertainty about relapse, treatment effects, and future medical plans. Clinicians should clearly explain that digital monitoring aims to identify clinically significant AF that could require changes in management, while avoiding unnecessary alarm about every rhythm irregularity [38].

Equity also matters, because older individuals, socioeconomically disadvantaged patients, people with limited health literacy, and those with poor internet access or low technological confidence may be less able to use smartwatches, mobile applications, or telehealth platforms effectively. A monitoring framework that relies exclusively on privately owned consumer technology risks favouring the most digitally proficient patients while marginalising those who may be at higher clinical risk. Realistic pathways include offering devices, facilitating training sessions, using mailed patch monitors, or initiating community-based ECG checks. Equity should be built into the pathway from the start [41,42,43,44].

Cost-effectiveness is uncertain because surveillance requires devices, staff time, data infrastructure, confirmatory ECGs, clinical appointments and downstream investigations. The estimate of approximately $57,900 per quality-adjusted life-year arose from modelling wearable AF screening in a general population aged 65 years or older [45]; it was not derived from patients with cancer, cardiac-surgery POAF, or competing bleeding and mortality risks. It should therefore be cited only as indirect economic evidence. Population-specific evaluation should include clinical workload, false alerts, anticoagulation changes, bleeding, stroke, readmission, oncology-treatment disruption, and digital exclusion.

These challenges mean that digital rhythm surveillance should be embedded in defined clinical pathways, and not left as device-led monitoring [38].

## 9. Clinical Interpretation and Decisions Potentially Supported by Monitoring

The following clinical consequences are plausible and testable, but have not been demonstrated in cancer survivors with POAF. Rhythm monitoring may document recurrence, but neither a low detected AF burden nor the absence of AF during a finite monitoring window has been validated as an independent criterion to initiate, continue, discontinue, or interrupt anticoagulation. Decisions should follow applicable AF and POAF guidance, while incorporating thromboembolic risk, dynamic bleeding risk, platelet count, tumour site, renal and hepatic function, treatment interactions, planned procedures, prognosis, and patient preferences [22,23,24,28,31]. CHA_2_DS_2_-VASc or CHA_2_DS_2_-VA and HAS-BLED may inform this assessment but should not determine it, particularly in active cancer, thrombocytopenia or POAF.

For biopsies, operations or periods of treatment-related thrombocytopenia, interruption and resumption of anticoagulation should be based on procedural bleeding risk, anticoagulant pharmacology, platelet count, renal function, and the underlying indication for anticoagulation. AF burden should not be used as a stand-alone justification. Monitoring results may prompt multidisciplinary reassessment, but no target-population study has shown that a low recorded burden makes interruption safe or that a higher burden mandates continuation.

Post-discharge ECG-confirmed AF should be distinguished from apparently transient index POAF and from pre-existing or established AF. Recurrent, symptomatic, or persistent AF could prompt cardiology or electrophysiology assessment for rate or rhythm control, including cardioversion or antiarrhythmic therapy where otherwise indicated. However, monitoring alone does not establish that intervention improves outcomes, and short asymptomatic episodes should not automatically trigger invasive or pharmacological treatment [22,23,24,31].

Detection of rapid AF could support earlier clinical review of symptoms, haemodynamics, ventricular function and existing rate-control therapy. Whether this approach improves medication adjustment, recovery, heart-failure outcomes or readmission compared with usual follow-up remains unknown and requires prospective evaluation [22,23,24].

Early outpatient detection could allow confirmatory ECG and clinical assessment before overt deterioration, but it could also increase emergency attendance and unnecessary investigation if every alert generates escalation. Claims that monitoring prevents readmission, facilitates cardioversion, or reduces healthcare use, should therefore be framed as hypotheses and measured directly in prospective studies [31,34,40].

Coordination with oncology is central because confirmed AF, anticoagulation and interacting cardiovascular medicines may affect procedural or systemic-treatment planning. Rhythm information may prompt multidisciplinary review, but reassuring monitoring data should not independently authorise cancer treatment to proceed, and an unconfirmed wearable alert should not delay or interrupt treatment. The effect of surveillance on cancer-treatment continuity remains unproven, and should be evaluated as an outcome [26,27,28,29].

## 10. A Proposed Digital Cancer-POAF Survivorship Pathway

**Figure 3:**
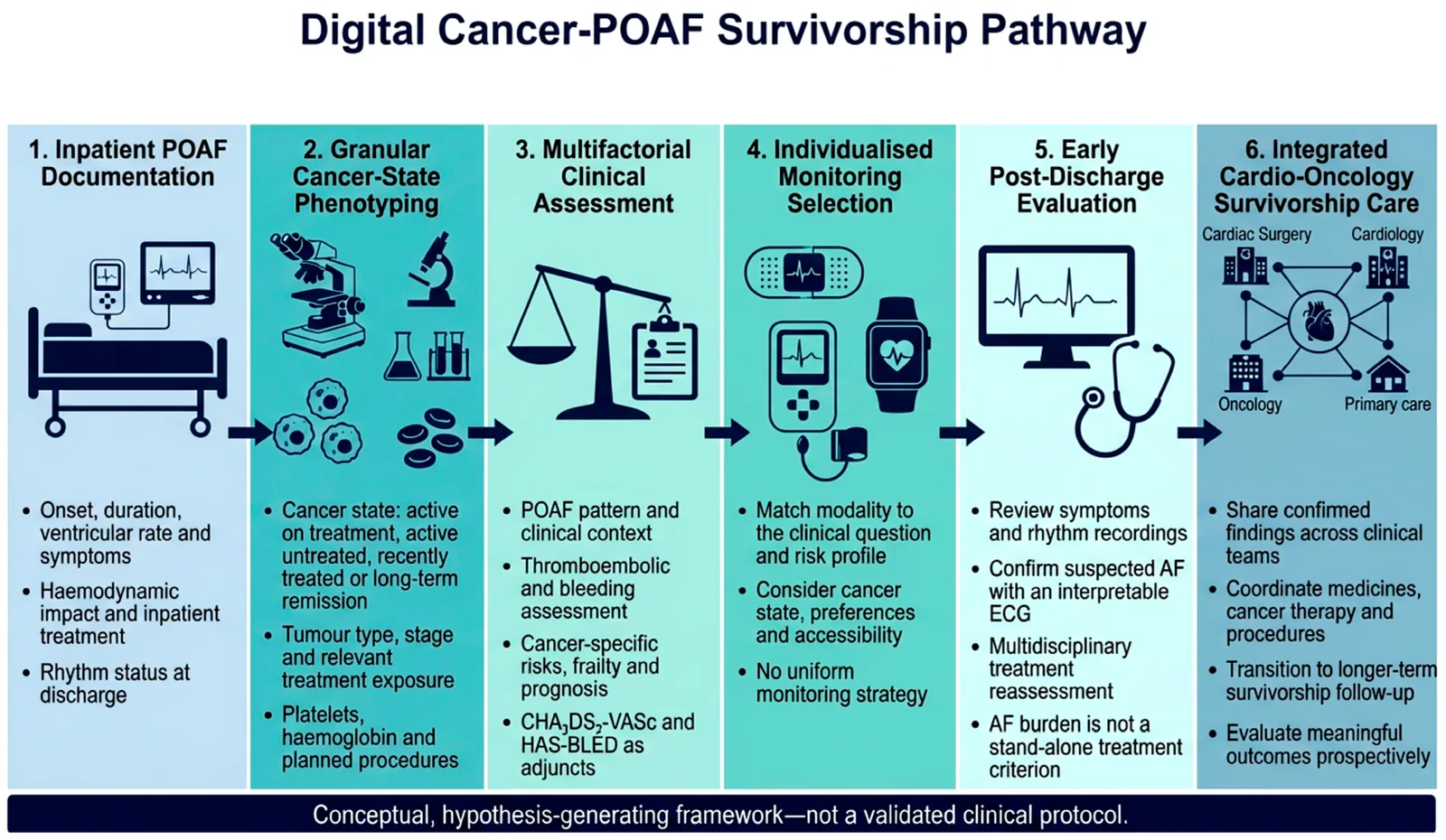
Proposed Digital Cancer-POAF Survivorship Pathway. This conceptual, hypothesis-generating framework comprises six stages: (1) inpatient POAF documentation; (2) granular cancer-state phenotyping; (3) multifactorial clinical assessment; (4) individualised monitoring selection; (5) early post-discharge evaluation; and (6) integrated cardio-oncology survivorship care. It is intended to organise post-discharge rhythm surveillance and multidisciplinary follow-up for prospective evaluation, not to mandate a uniform monitoring strategy or constitute a validated clinical protocol. AF, atrial fibrillation; ECG, electrocardiogram; CHA_2_DS_2_-VASc, stroke-risk score; HAS-BLED, bleeding-risk score; POAF, postoperative atrial fibrillation.

The Digital Cancer-POAF Survivorship Pathway, summarised in Figure 3, is the principal conceptual contribution of this review. Conventional POAF follow-up is generally organised around the cardiac-surgical episode, whereas cardio-oncology pathways commonly focus on treatment-related cardiotoxicity. This framework links the two by placing post-discharge rhythm surveillance within cancer-state phenotyping, anticoagulation reassessment, and multidisciplinary survivorship care [2,28,38]. It is intended to organise evidence gaps and generate testable hypotheses, not to mandate a uniform monitoring strategy or claim clinical effectiveness.

The pathway begins during the index cardiac surgical admission. Any occurrence of POAF should ideally be documented clearly before discharge, including timing of onset, duration, ventricular rate, symptoms, haemodynamic impact, and treatment given. This information should appear clearly in the discharge summary, and not be confined to inpatient records, as it identifies a patient who may require structured rhythm follow-up after hospitalisation [2,38].

Next, clinicians should assess the cancer phenotype using the categories in Table 1, rather than a present-or-absent label. Tumour type and stage, treatment exposure, thoracic radiotherapy, platelet count, haemoglobin, renal and hepatic function, frailty, prognosis and anticipated procedures may all modify the interpretation and potential value of monitoring [2,16,28,46].

Pre-discharge assessment could combine rhythm history and guideline-based stroke-risk evaluation with cancer-specific thrombotic and bleeding factors. Conventional scores do not fully incorporate active malignancy, thrombocytopenia, treatment interactions, or planned procedures, and should therefore be used as adjuncts rather than decisive algorithms [24,31,47,48].

The selection of the monitoring modality should be based on this comprehensive assessment of rhythm risk, cancer status, bleeding risk, life expectancy and functional status, patient preferences, and available local resources. A patient with active cancer, recurrent episodes of POAF, elevated thromboembolic risk, and acceptable functional status could be considered for extended patch monitoring or, in specific research contexts, an ILR. Conversely, a patient with a history of cancer and moderate risk may be better managed with a 14-day patch monitor or structured surveillance via wearable technology. In contrast, a patient with a remote history of low-risk cancer, transient self-terminating POAF, and minimal thromboembolic risk may benefit more from symptom education and selective ECG follow-up, instead of intensive digital monitoring [35,49].

Early post-discharge rhythm evaluation may be appropriate, but its timing and duration have not been validated for this population. Any suspected AF should be confirmed by an interpretable ECG before treatment decisions. Confirmed findings may prompt reassessment of anticoagulation, bleeding risk, symptoms, and rate or rhythm control; they should not automatically determine these decisions or the scheduling of oncology treatment [38,39].

The final stage involves coordination with oncology services and longer-term survivorship follow-up. Confirmed rhythm findings may prompt discussion of drug interactions, procedures, anticoagulation, and referral to cardio-oncology. However, the pathway has not been shown to prevent cancer-treatment delay, stroke, bleeding or readmission. It therefore treats POAF as a post-discharge risk signal and should be prospectively evaluated across cardiology, cardiac surgery, oncology and primary care [2,24].

## 11. Candidate Operational Protocol for Prospective Validation

To translate the conceptual stages shown in Figure 3 into a reproducible research design without presenting the pathway as established clinical care, Table 3 specifies one candidate operational protocol. Table 3 distinguishes evidence/guideline-informed principles from pragmatic author-proposed trial-design parameters. The rationale for early post-discharge ECG monitoring, ECG confirmation, and defined result ownership is evidence- or consensus-informed [15,22,24,38]. Exact monitoring windows, review intervals, alert thresholds, and stop-or-extend rules marked ^±^ are unvalidated research specifications, not clinical recommendations, and should not independently determine anticoagulation, rhythm-control treatment, or continuation of anticancer therapy.

## 12. Future Directions and Validation Outcomes

Because the pathway is hypothesis-generating, prospective multicentre studies should move beyond AF detection and evaluate stroke or systemic embolism, major bleeding, all-cause and cardiovascular mortality, heart failure, emergency attendance, readmission, and overall healthcare utilisation. Cancer-specific outcomes should include treatment delay or interruption, procedural disruption, and clinically relevant drug interactions. Patient-reported anxiety, acceptability, quality of life, and digital exclusion should also be measured [6,7,45].

Rhythm endpoints should include ECG-confirmed recurrence of at least 30 s as a diagnostic convention, alongside prespecified duration strata such as at least 6 min, 1 h and 24 h; longest episode; cumulative burden; episode count; symptom status; ventricular rate; and time to first recurrence. These categories are candidate reporting endpoints, not validated anticoagulation thresholds. Studies should test whether any burden measure adds clinically useful information beyond guideline-based and cancer-specific assessment [35,42].

Implementation outcomes should include technically adequate wear time, data completeness, false-positive alerts, confirmatory-ECG rates, alert volume, time to clinical review, staff workload, documentation quality, data-security incidents, and equity of access. Economic analyses should report device and personnel costs, downstream investigations, cost per confirmed case, and incremental cost per quality-adjusted life-year. These outcomes are essential because a system that increases detection while increasing workload, anxiety or inequity may not provide net clinical value [37,38,39,40,41,42,43,44,45,50].

Future studies should compare the complete framework with usual care and should use prespecified, clinically meaningful outcomes. Until such evidence exists, digital surveillance should be regarded as promising but unvalidated. Its results may support reassessment only after ECG confirmation and clinical interpretation; they should not function as automatic anticoagulation or cancer-treatment rules [2,28,38].

## 13. Limitations

This narrative review has several limitations. Evidence selection was not systematic and remains vulnerable to selection and citation bias, despite the reproducible updated search description. Only English-language publications were included. Definitions of POAF, AF episode duration, active cancer, previous cancer, and treatment timing vary across studies. Most evidence was extrapolated from general cardiac-surgery POAF, cancer-associated AF, consumer screening, or cryptogenic-stroke populations, rather than from the population targeted by the proposed pathway.

Risk scores have limited and heterogeneous validation in active cancer, and have not been validated as treatment algorithms for cancer-associated POAF. Digital devices, software and regulatory standards evolve rapidly, limiting the durability of modality-specific conclusions. Increased AF detection is a surrogate and does not establish reductions in stroke, bleeding, readmission or treatment disruption. No population-specific economic evaluation has tested this pathway. Finally, three cited publications originate from the same author group; those reports addressed prediction, mechanisms, and perioperative considerations, whereas the present review focuses on the distinct post-discharge surveillance question [2,3,4].

## 14. Conclusions

POAF in cancer survivors after cardiac surgery should not automatically be dismissed as a transient inpatient arrhythmia, but digital monitoring is not established as standard care in this population. The proposed “Digital Cancer-POAF Survivorship Pathway” is a hypothesis-generating framework that links cancer-state and treatment-specific assessment with post-discharge rhythm surveillance; it is not a validated clinical protocol. Prospective multicentre studies must determine whether structured surveillance improves stroke or systemic embolism, major bleeding, mortality, readmission and healthcare utilisation, cancer-treatment continuity, patient-reported outcomes and cost-effectiveness. Until such evidence is available, neither device-detected AF burden nor absence of AF during finite monitoring should independently determine anticoagulation or oncology-treatment decisions [2,21,37].

## Figures and Tables

**Table 1:** Clinically meaningful cancer-status categories for the proposed review framework.

Cancer State	Working Definition	Principal Modifiers Relevant to Surveillance
Active cancer receiving treatment	Current systemic anticancer therapy or radiotherapy.	Treatment class, drug interactions, cytopenias, renal/hepatic function, planned treatment cycles and procedures.
Active cancer not receiving treatment	Newly diagnosed, watch-and-wait, treatment temporarily withheld, or active disease without current therapy.	Tumour type and stage, prognosis, inflammatory/thrombotic activity, reason treatment is not being given, and planned interventions.
Recently completed treatment	A prespecified post-treatment interval; for example, up to 12 months in a future study, explicitly labelled as pragmatic.	Residual cardiotoxicity, recovery of platelet counts, recurrence risk, surveillance procedures, and time since the final treatment exposure.
Long-term remission or remote cancer history	No active malignancy or current anticancer treatment beyond the prespecified recent-treatment interval.	Late effects of thoracic radiotherapy or cardiotoxic therapy, cardiovascular comorbidity, functional status, and competing non-cancer risks.

Note: The categories organise clinical heterogeneity; they are not validated risk strata and should not be used as stand-alone treatment criteria.

**Table 2:** Comparison of digital rhythm-monitoring modalities.

Modality	Advantages	Limitations	Ideal Proposed Application
24–48 h Holter	Established continuous ECG; widely available.	Short window; low sensitivity for intermittent or delayed recurrence.	Frequent symptoms or an immediate short-term rhythm question.
Continuous ECG patch (commonly 7–14 days)	Longer continuous recording; relatively unobtrusive; quantifies episodes within the wear period.	Finite window, skin irritation, adherence and processing delay; does not prove absence of later AF.	Early post-discharge detection of asymptomatic or intermittent recurrence in research or selected care pathways.
Handheld single-lead ECG	Low burden, reusable and able to produce an ECG strip during symptoms.	Patient-initiated; limited assessment of asymptomatic AF or cumulative burden.	Intermittent symptoms or confirmation following a non-ECG alert.
Smartwatch ECG with or without PPG	Accessible to some patients; supports repeated spot ECGs and irregular-pulse alerts.	Digital and socioeconomic selection, intermittent ECG, artefact, commercial data silos and alert burden.	Longer-term patient-enabled surveillance when access, training and clinical review are available.
PPG-only application or sensor	Passive, scalable and low-burden.	Not diagnostic; vulnerable to motion, ectopy and false alerts; requires ECG confirmation.	Screening or triage to formal ECG assessment, not stand-alone diagnosis.
Implantable loop recorder	Continuous multi-year detection of infrequent events.	Invasive, expensive and may identify episodes of uncertain clinical significance.	Independent clinical indication or formal prospective research rather than routine POAF follow-up.

**Table 3:** Candidate operational specifications and evidence status for prospective evaluation of the Digital Cancer-POAF Survivorship Pathway.

Protocol Component	Candidate Prospective-Study Specification and Evidence Status
Entry criteria	Adults aged ≥18 years undergoing cardiac surgery who develop new-onset POAF before discharge, without documented preoperative AF, and who fall within one of the four cancer states defined in Table 1. POAF must be confirmed by a 12-lead ECG or interpretable telemetry tracing lasting ≥30 s ^‡^, or require treatment because of clinical or haemodynamic significance.
Baseline assessment	Eligibility and cancer-state phenotyping should be completed before discharge. Baseline documentation should include POAF onset, longest episode, ventricular rate, symptoms, treatment, discharge rhythm, platelet count, haemoglobin, renal and hepatic function, cancer therapy, planned procedures, and thromboembolic and bleeding factors.
Initial monitoring window	Early post-discharge ECG monitoring is evidence-informed [15]; the following timings are pragmatic candidate specifications. Monitoring would commence within 7 days ^†^ after discharge and continue for 14 days ^†^. Results would be reviewed within 7 days ^†^ after monitoring completion, with clinical follow-up at approximately 30 and 90 days ^†^.
Device selection	A 14-day ^†^ continuous ECG patch should be the default research modality for detecting asymptomatic recurrence. A handheld or smartwatch ECG may be used as an adjunct for symptom-triggered recordings where the patient has suitable access and ability. A 24–48 h Holter ^†^ may be used for frequent daily symptoms or an immediate short-term rhythm question. An implantable loop recorder should be limited to a nested research cohort or an independent clinical indication. PPG-only alerts should not be considered diagnostic.
ECG confirmation	Device-suspected or PPG-detected AF must be confirmed by an interpretable ECG tracing. ECG-confirmed AF lasting ≥30 s ^‡^ should constitute the principal recurrence endpoint, but should not be treated as an anticoagulation threshold.
Alert ownership and review	Defined ownership of monitoring results is consensus-informed [38]. A named cardiology rhythm service or study clinician would be responsible for receiving and reviewing recordings. For this candidate protocol, real-time alerts would be reviewed within one working day ^†^ and non-real-time patch reports within 7 days ^†^ of device return. Clinically relevant confirmed findings would be communicated to cardiac surgery, cardiology, oncology, and primary care.
Escalation criteria	Syncope, chest pain, severe breathlessness, hypotension, or another indication of haemodynamic instability warrants urgent clinical assessment [22,24]. The following are pragmatic candidate research triggers: ECG-confirmed AF accompanied by persistent symptoms, a ventricular rate ≥120 beats/min sustained for ≥30 min ^†^ or an episode lasting ≥24 h ^†^ would prompt same-day clinical review ^†^; other ECG-confirmed episodes would prompt multidisciplinary review within 72 h ^†^. These triggers would initiate reassessment only, and would not automatically determine treatment.
Stop or extend rules	The following are pragmatic candidate research rules. Monitoring would stop after 14 days ^†^ only when ≥10 analysable days ^†^ have been obtained, no AF has been detected and no unexplained symptoms remain. Monitoring would be repeated or extended for a further 14 days ^†^ when wear time is inadequate, recordings are inconclusive, symptoms occur without ECG documentation, or clinical suspicion remains high. Longer-term monitoring or an implantable recorder would be considered only within a prespecified research stratum or when independently clinically indicated.
Study outcomes	Studies should record ECG-confirmed recurrence, episode duration, cumulative AF burden, symptoms, time to first recurrence, confirmatory-ECG rates, treatment changes, stroke or systemic embolism, major bleeding, readmission, healthcare utilisation, cancer-treatment interruption, alert burden, and patient-reported outcomes.

Evidence status: Evidence/guideline-informed elements include early post-discharge ECG monitoring, ECG confirmation, non-diagnostic PPG alerts, defined result ownership and urgent assessment of haemodynamic instability [15,22,24,38]. ^†^ Pragmatic author-proposed research parameter; not validated in the cancer-POAF population or guideline-endorsed, and not an automatic treatment trigger. ^‡^ The ≥30 s duration is a research endpoint/eligibility convention, not an anticoagulation or treatment threshold [22,24].

## Data Availability

No new data were created or analysed in this study. Data sharing is not applicable to this article.

## Abbreviations

The following abbreviations are used in this manuscript:

AF	Atrial fibrillation
AI	Artificial intelligence
BTK	Bruton’s Tyrosine Kinase
CHA_2_DS_2_-VASc	Congestive heart failure, hypertension, age ≥75 years (2 points), diabetes mellitus, stroke/transient ischaemic attack/systemic embolism (2 points), vascular disease, age 65–74 years, and sex category
ECG	Electrocardiogram
EHR	Electronic health record
FHIR	Fast Healthcare Interoperability Resources
GDPR	General Data Protection Regulation
HAS-BLED	Hypertension, Abnormal renal/liver function, Stroke, Bleeding, Labile INR, Elderly, Drugs/alcohol—bleeding-risk score
HIPAA	Health Insurance Portability and Accountability Act
ILR	Implantable loop recorder
OR	Odds ratio
POAF	Postoperative atrial fibrillation
PPG	Photoplethysmography
